# Supplementary material for: The Systems Biology Research Tool: evolvable open-source software
Source: BMC Syst Biol. 2008 Jun 29;2:55. doi: 10.1186/1752-0509-2-55 (PMC2446383; doi:10.1186/1752-0509-2-55)
Supplement: Additional file 1 — SBRT Archive. An archive of the current version of the Systems Biology Research Tool. [file 1752-0509-2-55-S1.zip › sbrt-1.4.0/doc/developers_guide/api/sbrt/kernel/fba/comp/class-use/InitialPointComputer.Results.html]

Uses of Class sbrt.kernel.fba.comp.InitialPointComputer.Results


|  |  |  |
| --- | --- | --- |
| **Overview** \| **Package** \| **Class** \| **Use** \| **Tree** \| **Deprecated** \| **Index** \| **Help** | |  |
| PREV   NEXT | **FRAMES**    **NO FRAMES**     **All Classes** |  |


---


## **Uses of Class sbrt.kernel.fba.comp.InitialPointComputer.Results**

| Packages that use InitialPointComputer.Results | |
| --- | --- |
| **sbrt.kernel.fba.comp** | Provides classes and interfaces for performing Flux Balance Analysis computations. |
| **sbrt.kernel.fba.proc** | Provides classes and interfaces for executing Flux Balance Analysis processes. |

Uses of InitialPointComputer.Results in sbrt.kernel.fba.comp

| Methods in sbrt.kernel.fba.comp that return InitialPointComputer.Results | |
| --- | --- |
| `InitialPointComputer.Results` | `InitialPointComputer.run()`             Computes an initial point, or flux vector, in the flux space of the fluxome used by this computer. |

Uses of InitialPointComputer.Results in sbrt.kernel.fba.proc

| Method parameters in sbrt.kernel.fba.proc with type arguments of type InitialPointComputer.Results | |
| --- | --- |
| `void` | `InitialPointGenerator.run(ResultsManager<InitialPointComputer.Results> resultsManager)`             Generates an initial point and adds it to the provided results manager. |
| `void` | `SvInitialPointProcess.run(ResultsManager<InitialPointComputer.Results> resultsManager)`             Runs this process and adds the generated results to the provided results manager via its `ResultsManager.addData(Object)` method. |

---


|  |  |  |
| --- | --- | --- |
| **Overview** \| **Package** \| **Class** \| **Use** \| **Tree** \| **Deprecated** \| **Index** \| **Help** | |  |
| PREV   NEXT | **FRAMES**    **NO FRAMES**     **All Classes** |  |


---
